# Supplementary material for: An Integrated Patient-Centred Medical Home (PCMH) Care Model Reduces Prospective Healthcare Utilisation for Community-Dwelling Older Adults with Complex Needs: A Matched Observational Study in Singapore
Source: Int J Environ Res Public Health. 2023 Sep 27;20(19):6848. doi: 10.3390/ijerph20196848 (PMC10572627; doi:10.3390/ijerph20196848)
Supplement: Supplementary file 1 [file ijerph-20-06848-s001.zip › ijerph-2376225-supplementary.pdf]

Supplementary Materials

S1. Descriptive analysis for healthcare utilisation (intervention group n=165)

Quarter: Refers to a 3-month period, e.g., 4th quarter refers to the fourth 3-month period post-enrolment date in PCMH for the intervention group.

**Utilisation for each health service**	**Pre-enrolment**	**Post-enrolment**				
	**One quarter before enrolment**	**1st quarter**	**2nd quarter**	**3rd quarter**	**4th quarter**	**5th quarter**
**No. of all-cause acute hospitalisations**						
**Pooled (n=165)**						
Mean (sd)	0.11 (0.37)	0.10 (0.30)	0.10 (0.30)	0.12 (0.39)	0.11 (0.38)	0.10 (0.36)
Median (IQR)	0 (0-0)	0 (0-0)	0 (0-0)	0 (0-0)	0 (0-0)	0 (0-0)
Range (min-max)	0-2	0-1	0-1	0-2	0-3	0-2
**Only among utilised** n (%)	15 (9.1%)	16 (9.7%)	17 (10.3%)	15 (9.1%)	15 (9.1%)	12 (7.27%)
Mean (sd)	1.2 (0.41)	1.0 (0.001)	1.0 (0.001)	1.3 (0.46)	1.2 (0.56)	1.25 (0.45)
Median (IQR)	1 (1-1)	1 (1-1)	1 (1-1)	1 (1-2)	1 (1-1)	1 (1-1.5)
Range (min-max)	1-2	1-1	1-1	1-2	1-3	1-2
**No. of emergency department visits**						
**Pooled (n=165)**						
Mean (sd)	0.16 (0.55)	0.15 (0.41)	0.15 (0.39)	0.19 (0.50)	0.12 (0.39)	0.08 (0.32)
Median (IQR)	0 (0-0)	0 (0-0)	0 (0-0)	0 (0-0)	0 (0-0)	0 (0-0)
Range (min-max)	0-4	0-2	0-2	0-2	0-3	0-2
**Only among utilised** n (%)	18 (10.9%)	22 (13.3%)	22 (13.3%)	23 (13.9%)	16 (9.7%)	11 (6.67%)
Mean (sd)	1.4 (0.98)	1.14 (0.35)	1.09 (0.29)	1.35 (0.49)	1.19 (0.54)	1.18 (0.40)
Median (IQR)	1 (1-1)	1 (1-1)	1 (1-1)	1 (1-2)	1 (1-1)	1 (1-1)
Range (min-max)	1-4	1-2	1-2	1-2	1-3	1-2
**No. of specialist outpatient clinic visits**						
**Pooled (n=165)**						
Mean (sd)	1.47 (2.18)	1.56 (2.72)	1.96 (4.46)	1.36 (2.34)	1.29 (2.33)	0.95 (1.88)
Median (IQR)	0 (0-2)	0 (0-3)	0 (0-2)	0 (0-2)	0 (0-2)	0 (0-1)
Range (min-max)	0-10	0-18	0-44	0-14	0-14	0-13
**Only among utilised** n (%)	72 (43.6%)	72 (43.6%)	71 (43.0%)	69 (41.8%)	61 (37.0%)	46 (27.88%)
Mean (sd)	2.53 (2.65)	3.57 (3.13)	4.55 (5.88)	3.26 (2.62)	3.49 (2.65)	3.20 (2.17)
Median (IQR)	2 (0-4)	3 (2-4)	3 (1-5)	3 (2-4)	3 (2-4)	3 (2-4)
Range (min-max)	0-10	1-18	1-44	1-14	1-14	1-13
**No. of public primary care (polyclinic) visits**						
**Pooled (n=165)**						
Mean (sd)	0.61 (1.03)	0.33 (0.77)	0.22 (0.57)	0.22 (0.64)	0.24 (0.72)	0.21 (0.72)
Median (IQR)	0 (1-2)	0 (0-0)	0 (0-0)	0 (0-0)	0 (0-0)	0 (0-0)
Range (min-max)	0-5	0-3	0-3	0-4	0-5	0-6

**Only among utilised** n (%)	54 (32.7%)	30 (18.2%)	24 (14.6%)	23 (13.9%)	24 (14.6%)	18 (10.91%)
Mean (sd)	1.87 (0.93)	1.80 (0.76)	1.50 (0.59)	1.61 (0.84)	1.63 (1.17)	1.78 (1.31)
Median (IQR)	2 (1-2)	2 (1-2)	1 (1-2)	1 (1-2)	1 (1-2)	1 (1-2)
Range (min-max)	1-5	1-3	1-3	1-4	1-5	1-6
